# Primary Treatment Effects for High-Grade Serous Ovarian Carcinoma Evaluated by Changes in Serum Metabolites and Lipoproteins

**DOI:** 10.3390/metabo13030417

**Published:** 2023-03-12

**Authors:** Cecilie Fredvik Torkildsen, Marie Austdal, Ann-Charlotte Iversen, Tone Frost Bathen, Guro Fanneløb Giskeødegård, Elisabeth Berge Nilsen, Grete Alræk Iversen, Ragnar Kvie Sande, Line Bjørge, Liv Cecilie Vestrheim Thomsen

**Affiliations:** 1 Department of Obstetrics and Gynecology, Stavanger University Hospital, 4068 Stavanger, Norway; cecilie.torkildsen@uib.no (C.F.T.);; 2 Centre for Cancer Biomarkers CCBIO, Department of Clinical Science, University of Bergen, 5020 Bergen, Norway; 3 Department of Research, Stavanger University Hospital, 4068 Stavanger, Norway; 4 Centre of Molecular Inflammation Research (CEMIR), Department of Clinical and Molecular Medicine, Norwegian University of Science and Technology (NTNU), 7491 Trondheim, Norway; 5 Department of Gynecology and Obstetrics, St. Olavs Hospital, Trondheim University Hospital, 7006 Trondheim, Norway; 6 Department of Circulation and Medical Imaging, Norwegian University of Science and Technology, 7491 Trondheim, Norway; 7 K.G. Jebsen Center for Genetic Epidemiology, Department of Public Health and Nursing, Norwegian University of Science and Technology (NTNU), 7491 Trondheim, Norway; 8 Department of Obstetrics and Gynecology, Haukeland University Hospital, 5021 Bergen, Norway; 9 Department of Clinical Science, University of Bergen, 5020 Bergen, Norway

**Keywords:** HGSOC, metabolomics, metabolites, lipoproteins, phenotypes, cytoreductive surgery, precision medicine, personalized medicine

## Abstract

High-grade serous ovarian carcinoma (HGSOC) is the most common and deadliest ovarian cancer subtype. Despite advances in treatment, the overall prognosis remains poor. Regardless of efforts to develop biomarkers to predict surgical outcome and recurrence risk and resistance, reproducible indicators are scarce. Exploring the complex tumor heterogeneity, serum profiling of metabolites and lipoprotein subfractions that reflect both systemic and local biological processes were utilized. Furthermore, the overall impact on the patient from the tumor and the treatment was investigated. The aim was to characterize the systemic metabolic effects of primary treatment in patients with advanced HGSOC. In total 28 metabolites and 112 lipoproteins were analyzed by nuclear magnetic resonance (NMR) spectroscopy in longitudinal serum samples (n = 112) from patients with advanced HGSOC (n = 24) from the IMPACT trial with linear mixed effect models and repeated measures ANOVA simultaneous component analysis. The serum profiling revealed treatment-induced changes in both lipoprotein subfractions and circulating metabolites. The development of a more atherogenic lipid profile throughout the treatment, which was more evident in patients with short time to recurrence, indicates an enhanced systemic inflammation and increased risk of cardiovascular disease after treatment. The findings suggest that treatment-induced changes in the metabolome reflect mechanisms behind the diversity in disease-related outcomes.

## 1. Introduction

Epithelial ovarian cancer (EOC) is the third most common gynecologic malignant tumor [1]. The most frequent and revised EOC subtype is high-grade serous ovarian carcinoma (HGSOC). Despite advances in medical and surgical treatment, the overall prognosis remains poor [2,3,4]. Although most patients with late-stage disease respond to the initial treatment with surgery combined with chemotherapy, more than 80% of the patients will experience recurrence and ultimately death [5,6]. It is challenging to predict the individual outcome as the disease is marked by a heterogeneous biology, and patients with similar stage, grade, and histology experience differences in treatment responses and prognosis [7,8,9,10].

Metabolism is essential for every cell function and provides energy for vital processes such as cell division, growth, and differentiation. The essential production of energy changes with the development of diseases, and while normal cells provide energy through mitochondrial oxidative phosphorylation, malignant cells, such as those present in EOC, can develop alternative approaches for energy production based on their unique microenvironments [11]. This metabolic reprogramming is recognized as a cancer hallmark [12]. Malignant cells seem to adapt the energy production to overcome their nutrient-deficient environment, increase their resilience to treatment, and develop a more favorable microenvironment for tumor prosperity and growth [11]. The disease adaptations and subsequent heterogeneity are reflected through the metabolic profile [13]. The glycolytic activity seems to be higher in the most aggressive histologic EOC subtypes [14], and cellular glycogen accumulation is a feature observed in the chemo-resistant subtype clear cell ovarian cancer [15]. The fatty acid-induced metabolic reprogramming of the tumor microenvironment (TME) that results in alterations in the tumor immune responses has been described in preclinical EOC models [16].

The metabolic signatures in blood, plasma, ascitic fluid, and urine have been suggested as biomarkers for detection and diagnostics as well as to guide targeted therapy for HGSOC patients [17,18,19]. Additionally, measurements of lipoprotein subfractions, the main carriers of triglycerides and cholesterol in the blood stream, provide extensive information on metabolic activity and may be reflective of an individual’s health status. Data from clinical treatment trials in other solid tumors signify that the metabolite and lipoprotein composition in blood can predict the response to therapy, prognosis, and long-term risk for cardiovascular disease [20,21]. How the treatment regimens recommended to patients with HGSOC influence their metabolic balance is still unclear [22]. This study aimed to improve the understanding of the metabolic changes caused by cancer treatment and the pathophysiological processes that contribute to the treatment outcomes in HGSOC patients. Consequently, we have explored the longitudinal changes in circulating metabolite and lipoprotein composition during the initial treatment of primary advanced HGSOC utilizing the nuclear magnetic resonance (NMR) of biofluids.

## 2. Materials and Methods

### 2.1. Study Design and Patients

**Figure 1:**
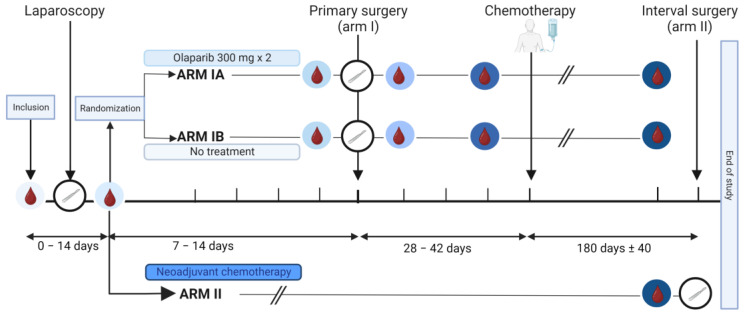
Graphical illustration of the IMPACT trial. The time points of the collection of serum samples are marked with blood drop symbols in circles with different shades of blue, and the time points of the surgeries are indicated by white circles containing a scalpel. Arm I included patients eligible for primary cytoreductive surgery (Predictive Index Value [PIV] score < 8). They were randomized into Arm IA or Arm IB. Patients in Arm IA, but not Arm IB, received an oral administration of olaparib tablets 300 mg × 2 for 7–14 days prior to the primary surgery. Patients in Arm II were laparoscopically evaluated to be inoperable (PIV ≥ 8) and received three rounds with neoadjuvant chemotherapy. Subgroups compared for serum analysis are shown in supplementary material (Figure S1). Created with BioRender.com.

The IMPACT trial is an open-label window-of-opportunity study in patients newly diagnosed with presumed advanced stage HGSOC. The trial was conducted at the Department of Obstetrics and Gynecology at Haukeland University Hospital, Bergen, Norway and Stavanger University Hospital, Stavanger, Norway. Included patients had to be eligible for a laparoscopic tumor assessment to determine whether complete tumor resection was feasible [23,24]. Furthermore, only patients with an Eastern Cooperative Oncology Group (ECOG) performance status below 3 and adequate bone marrow, liver, and renal functions [24,25] could be included. Key exclusion criteria were significant medical illnesses or conditions that would limit the possibility for surgical treatment. Patients were stratified to primary cytoreductive surgery followed by adjuvant chemotherapy (Arm I) or neoadjuvant chemotherapy (NACT) eventually followed by interval surgery if feasible (Arm II) (Figure 1). The stratification was based on a structured laparoscopic evaluation by two surgeons based on Predictive Index Value (PIV) scores previously reported by Fagotti et al. [23]. Patients with a PIV score < 8 were offered primary cytoreductive surgery and allocated to Arm I. Patients with a PIV score ≥ 8 were selected for the initiation of NACT and allocated to Arm II. Patients in Arm II received NACT according to the national guidelines, that is, three cycles of carboplatin and paclitaxel, before they were evaluated for interval surgery [26]. The last study visit for Arm II participants was either on the day of the tumor reductive interval surgery or at the time of evaluation after three cycles if operation at that point was not a feasible option. Participants allocated to Arm I after the laparoscopic procedure were randomized using a sealed envelope system to either 7–14 days of treatment with olaparib, an inhibitor of the enzyme poly-ADP-ribose polymerase (PARP) (Arm IA), or no treatment prior to the cytoreductive surgery (Arm IB). Patients with histopathological diagnoses other than HGSOC were excluded.

### 2.2. Assessments

Clinical and biochemical evaluation were performed for all participants at baseline, and after laparoscopy. For patients in Arm I, clinical examinations and biochemical analyses were done before and after primary cytoreductive surgery, before the initiation of chemotherapy, and at the last follow-up visit after three cycles of chemotherapy. In Arm II, the evaluations were performed at baseline, after laparoscopy, and after three rounds of NACT treatment before potential interval surgery. A computerized tomography (CT) scan was included in the evaluation prior to the laparoscopy and as part of the follow-up after three chemotherapy cycles. The tumor response was assessed according to Response Evaluation Criteria in Solid Tumors (RECIST) version 1.1 and by the tumor marker CA125 [27]. Toxicities were monitored until 30 days after the completion of treatment or removal from the study, or until death, whichever occurred first. Common Terminology Criteria for Adverse Events (CTCAE), version 4.0 was utilized to grade adverse events. Progression-free survival (PFS) was calculated from the date of diagnosis until the date the progression of disease was determined. Standard-of-care treatment and follow-up were recommended for the participants after the completion of the trial period. Overall survival (OS) was defined as the period from the date of diagnosis until death of any cause.

### 2.3. Specimen Collection

Serum samples (*n* = 112) were collected in SSTII plus advanced vacutainers of 8.5 mL and left to clot for 30–90 min at room temperature before centrifugation at 1800× *g* for 10 min. Serum aliquots of 1–2 mL were stored at –80 °C until analysis. All samples, except for those collected after the diagnostic laparoscopy, were non-fasting. Routine laboratory analyses of CA125, albumin, lymphocyte count, total cholesterol, high-density lipoprotein (HDL), triglyceride, low-density lipoprotein (LDL), and creatinine were measured by accredited methods at the Department of Clinical Chemistry in Stavanger University Hospital, Stavanger, Norway and Haukeland University Hospital, Bergen, Norway.

### 2.4. Metabolomics Analyses

The metabolomics analyses were performed at the MR Core Facility at the Norwegian University of Science and Technology (NTNU), Trondheim, Norway. For the serum analyses, 350 µL serum was thawed, mixed with a 350 µL buffer (20% D_2_O with 0.075 M Na_2_HPO_4_, 6.2 mM NaN, 4.6 mM triple super phosphate [TSP], pH 7.4) before 550 µL of the mix was transferred to 3 mm NMR tubes. Two quality control samples were prepared and analyzed from anonymous donors. The tubes were immediately transferred to the NMR spectrometer for analysis (Bruker Avance III 600 MHz spectrometer [Bruker Biospin GmbH, Ettlingen, Germany]). The experiments were fully automated using the SampleJet^TM^ with Icon–NMR on TopSpin 3.1 software (Bruker Biospin). Carr–Purcelli–Meiboom–Gill and nuclear Overhauser effect spectroscopy spectra were acquired with water suppression at a temperature of 37 °C. The free induction decays were Fourier transformed into 128k real data points after 0.3 Hz exponential line broadening. Spectral data were further preprocessed in Matlab R2020b (The Mathworks Inc., Natick, MA, USA). The spectra were referenced to the left peak of the alanine doublet at 1.47 ppm [28]. The region of interest was defined as (0.1–9.2 ppm), excluding the water region (4.3–5.1 ppm). Metabolite peaks were identified using the human metabolome database, published literature, and in-house previously assigned spectral peaks in serum. Metabolites were quantified by integrating fixed spectral regions corresponding to each peak and adjusting for T2 relaxation times [29]. For metabolites with more than one available peak, the proton-weighted average of the peaks was calculated and used for further analyses. The concentration of glucose was set equal to the automatically quantified glucose concentration (Bruker B.I. Quant–PS^TM^ system [30]) and remaining metabolites scaled accordingly to obtain absolute metabolite concentrations. For one patient, the acetate concentration was imputed by k-nearest neighbors using impute v.1.72.2 in R (k = 10) due to an excessively high metabolite level. Lipoproteins in serum were quantified using the commercial Bruker IVDr Lipoprotein Subclass analysis (B.I.LISA^TM^) method from Bruker BioSpin. This method yields 112 quantitative lipoprotein parameters: the concentrations of lipids (cholesterol [CH], free cholesterol [FC], triglycerides [TG], and phospholipids [PL]) in serum and in four main lipoprotein classes: VLDL, IDL, LDL, and HDL as well as 15 subclasses (VLDL 1–5, LDL 1–6, and HDL 1–4). Simultaneously, the concentrations in serum of apolipoproteins (Apo-A1, Apo-A2, and Apo-B) and two main classes (HDL and LDL) and 10 subclasses (HDL 1–4 and LDL 1–6) of lipoprotein subfractions are quantified. The model also returns 12 calculated parameters, including ratios of LDL–CH/HDL–CH and Apo-B/Apo-A1 and 10 particle numbers (particle numbers of total serum, VLDL, IDL, LDL, and LDL 1–6). A total of 28 metabolites and 112 lipoprotein parameters were quantified (Figure S2).

### 2.5. Statistical Methods

Multiple testing correction for metabolite and lipoprotein analyses was performed with the Benjamini–Hochberg procedure, and adjusted *p*-values < 0.05 were considered significant [31]. PFS was divided into long and short PFS based on the median value. Two patients were excluded due to early demise (<60 days after surgery) and because of short (6 months) follow-up time without disease relapse.

Linear mixed effect models (LMM) were used to assess changes from baseline measurements [32] to the measurements at each of the subsequent time points. For the assessment of longitudinal changes, patients were examined based on which treatment they were given prior to surgery, the surgical outcome, and their PFS. For the subgroup analysis, the formulas for the linear mixed model were used: (lme): biomarker ~ time + group + time:group + (1|ID) and (lme): biomarker ~ time + (1|ID) for time-dependent changes [33]. *p*-values from the LMM were calculated using the Satterthwaites method using the lmertest package in R.

A heatmap of the metabolites and lipoprotein subfractions measured at inclusion was constructed using the package pheatmap v.1.0.12 in R. Each variable was mean centered and variance scaled across samples so that a higher value indicates relatively higher concentration among the included samples. Variables and samples were both clustered using Euclidian distance and complete linkage.

Repeated measures ANOVA simultaneous component analysis+ (RM–ASCA+) was used for multivariate analysis to visualize metabolic changes occurring over time in the different groups [34,35]. The RM–ASCA+ allows an overall separation of time effects from group effects. The method extends repeated measures LMM to the multivariate case by first decomposing the multivariate response matrix into effect matrices according to the specified LMM. The resulting effect matrices are then analyzed by principal component analysis (PCA), and the results are summarized into PCA scores and loadings. PC1 is the line that best accounts for the shape of the points and represents the maximum variance direction in the data. Positive loadings indicate a variable and a principal component that are positively correlated: an increase in one indicates an increase in the other. Large (either positive or negative) loadings indicate that a variable has a strong effect on that principal component. The above-mentioned LMM were used in the RM–ASCA+ analysis (Tables S3–S5). Non-parametric bootstrapping was used to construct 95% confidence intervals for the scores and loadings. Bootstrapping was performed by resampling until original sample size was achieved, and the process was repeated 1000 times. The 2.5th and 97.5th percentiles of the bootstrapped estimates were used as the lower and upper bounds for the intervals. LMM and RM–ASCA+ analysis were performed in R using the lme4 v1.1–31 [33] and ALASCA v.1.0.0 libraries [35]. Additionally, Orthogonal Partial Least-Squares Discriminant Analysis (OPLS–DA) analyses were performed in metabolite and lipoprotein composition in order to unravel the group-related variation of the measured data between Arm I and Arm II [36].

Differences in clinical variables between patient groups were assessed by *t*-tests for continuous variables and chi-squared tests and ANOVA for categorical variables. Kaplan–Meier curves were used to illustrate PFS. Statistical analyses were performed in SPSS v. 26 (SPSS, Chicago, IL, USA), GraphPad Software (San Diego, CA, USA), and R [37].

### 2.6. Ethics and Approvals

The study protocol and clinical trial set-up were approved by the Regional Ethical Committee of Norway (REKVest 2017/1168) and the Norwegian Medicine Agency (17/10642), and the trial was registered at Clinicaltrials.gov (NCT03378297) and the European Union Drug Regulating Authorities Clinical Trials Database (EudraCT 2017–001689–11). The study was conducted in accordance with the protocol, good clinical practice guidelines, and provisions of the Declaration of Helsinki and all local regulations. All subjects provided written informed consent before inclusion.

## 3. Results

### 3.1. Clinical Characteristics

A total of 26 women were enrolled in the trial. Two patients were excluded based on the results from the histopathological evaluation of tissues biopsied after the laparoscopy. Overall, 15 patients were included in Arm I to primary cytoreductive surgery (five in Arm IA, ten in Arm IB) and nine patients in Arm II for the immediate initiation of NACT (Figure 1). The patients in Arm I were younger than in Arm II at diagnosis (64.2 years vs. 67.3 years, *p* = 0.045) but comparable with regards to BMI (mean 25, range 18–32 kg/m^2^, both arms, *p* = 0.404) and mean CA125 levels (897 and 837 KU/L in Arm I and Arm II, respectively, *p* = 0.471) (Table S1). BMI was registered in 19/24 patients throughout the study and revealed overall stable BMI values. The majority (16/19) had a lower BMI at the end of treatment. Six patients were using cholesterol-lowering drugs at inclusion, equally distributed between the study arms (4/15 in Arm I and 2/9 in Arm II). The follow-up time varied from six months up to four years. The follow-up analysis after the study period revealed that 19 of the patients had experienced relapse of the disease (11/15 in the primary surgery group and 8/9 in the neoadjuvant group). Nine of the patients had died during the follow-up period (3/15 in Arm I and 5/9 in Arm II).

**Figure 2:**
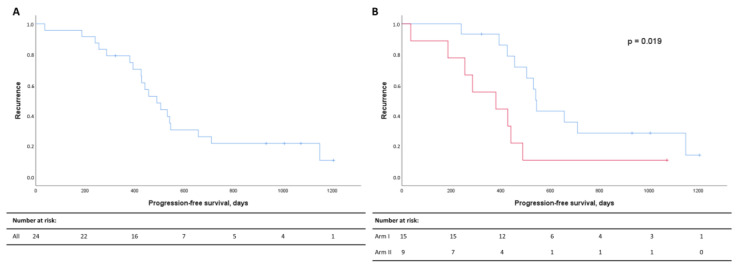
Kaplan–Meier curves for progression-free survival (PFS) for all patients (**A**) and for Arm I (blue) and Arm II (red) (**B**). PFS in days after diagnosis are listed on the x-axis, and the cumulative probabilities of recurrence in the cohort are shown along the y-axis. Censored data are indicated by small vertical lines. Numbers at risk across time are listed in the table below the Kaplan–Meier curves.

The median PFS for the whole patient cohort demonstrated that 50% had progressed 497 days after diagnosis (Figure 2A). Recurrence rates were significantly higher in Arm II (Figure 2B).

### 3.2. Serum Profiling of All HGSOC Patients at Inclusion

**Figure 3:**
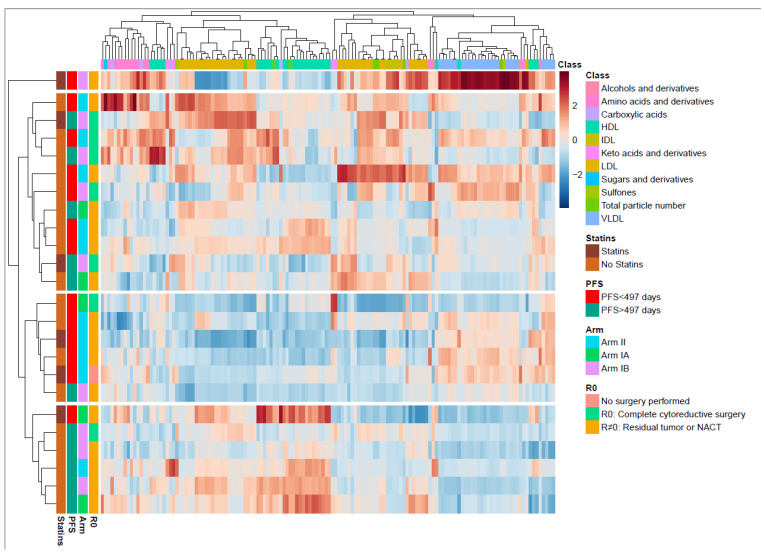
Overview of the metabolite and lipid composition in the total cohort at inclusion. Heatmap with unsupervised hierarchical clustering demonstrating the composition of metabolites and lipids. Each row represents one patient. The blocks to the left are color coded according to the different relevant subgroups (right). The metabolite and lipoprotein classes are shown at the top. Color scale represents a lower (blue) or higher (red) concentration of a metabolite or lipoprotein subfraction. The middle-boxed area in the heatmap indicates a clustering of patients with short progression-free survival (PFS), the majority with residual tumor. The lower boxed area shows a clustering of patients with long PFS, the majority with residual tumor. The color scale is relative and scaled for inter-individual differences between the patients for each metabolite subfraction. PC, principal component; PFS, progression-free survival; HDL, high density lipoproteins; IDL, intermediate dense lipoproteins; LDL, low density lipoproteins; VLDL, very low density lipoproteins.

To better understand the metabolic changes caused by the cancer disease and cancer treatment, the longitudinal changes in the metabolite and lipoprotein composition were examined. When the circulating metabolites and lipoprotein subfraction profiles were compared at inclusion, no differences were identified between the patients in Arm I and in Arm II. At inclusion, we see a widespread distribution of metabolites and lipoprotein subfractions in a hierarchically clustered heatmap annotated by clinical outcomes (Figure 3). Four main clusters were found, of which one consisted of only one patient who demonstrated greatly increased VLDL, and one demonstrated no particular clinical subtypes associated with metabolic patterns. However, the two other clusters demonstrated a group of patients (n = 5/6) with short PFS (<497 days) and one of patients (n = 5/6) with long PFS. The short PFS group had higher serum subfractions of VLDL and lower HDL lipoproteins (Figure 3), while the long PFS group had increased serum subfractions of HDL and lower VLDL. The patterns of VLDL and HDL differences were analyzed in detail by RM–ASCA+ (Table S4).

### 3.3. Treatment Effects on the Longitudinal Development of Metabolites and Lipoproteins

**Figure 4:**
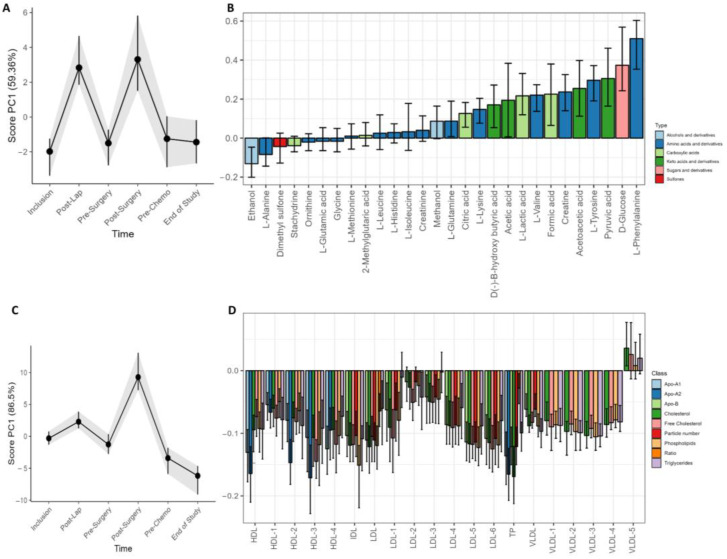
Longitudinal development of circulating metabolites and lipoprotein subfractions of all patients during the study period. Results show scores and loadings from RM–ASCA+ analysis for (**A**) circulating metabolites and (**C**) lipoprotein subfractions (n = 23/24). The response to increases or decreases of the trajectory lines is explained by their corresponding loading plots (**B**,**D**). The loading plots (**B**,**D**) describe the contribution of each metabolite or lipoprotein (x-axis) to their corresponding score (y-axis). The scores show the overall development of the patients over time. An increase in the PC1 score (**A**,**C**) indicates an increase in metabolite or lipoprotein subfractions with positive loadings and reduced negatively loaded subfractions and/or subfractions with decreasing negative loading. A decrease in PC1 score indicates a decrease in metabolite or lipoprotein subfractions with positive loadings but an increase in the subfractions with negative loadings. The 95% confidence interval is shown with black lines within the bars.

Changes in metabolite and lipoprotein distribution over time were examined by LMM and RM–ASCA+ (Figure 4). The surgical procedures were followed by a strong and transient adjustment of metabolite and lipoprotein subfractions in serum. Interestingly, the variation was stronger for metabolites than lipoproteins in response to laparoscopy, while the primary cytoreductive surgery resulted in a general increase in circulating metabolites and lipoprotein subfractions (Figure 4A,C). The lipoprotein loading plot (Figure 4C) demonstrates higher PC1 scores at the post surgery visit but not after the diagnostic laparoscopy. Several metabolite subfractions were increased after both surgical procedures, including phenylalanine, glucose, pyruvic acid, tyrosine, acetoacetic acid, creatine, formic acid, valine, lactate, acetic acid, β-hydroxybutyric acid, and lysine. Slightly lower levels of ethanol and alanine was also observed. The LMM analysis at the post surgery visit demonstrated overall higher subfractions of phenylalanine (*p* < 0.001), glucose (*p* < 0.001), tyrosine (*p* < 0.001), and pyruvate (*p* = 0.040). The laparoscopy led to an increase in valine (*p* = 0.002), lysine (*p* = 0.003), glutamine (*p* = 0.008), lactic acid (*p* = 0.016), and formic acid (*p* = 0.038), but no significant lipoprotein changes. The cytoreductive surgery led to an increase of creatine (*p* < 0.001) from baseline, and the lipoprotein subfractions were modified with decreased levels of 42 lipoprotein subfractions together with increased levels of four VLDL5 subfractions (V5TG, *p* = 0.02; V5PL, *p* = 0.02; V5CH, *p* < 0.001; V5FC, *p* < 0.001, all from LMM).

At the end of the study, the patients demonstrated a general increase in all lipoprotein subfractions except for VLDL5. In the LMM analysis, 16 lipoprotein subfractions were elevated, including total cholesterol (TPCH) and Apolipoprotein-A1 and A2 (TPA1 and TPA2) as well as Apo-A1 HDL, total HDL, phospholipids in IDL, and triglycerides in IDL. The elevated subfractions included in addition seven elevated HDL subfractions and two VLDL subfractions (Table S3).

Large effects or changes not related to the strong influence of surgery were explored by performing RM–ASCA+ analysis without the two post-surgery visits. The longitudinal metabolite analysis revealed an increase from inclusion to the end of study of the circulating amino acids alanine, isoleucine, histidine, leucine, methylglutarate, tyrosine, glutamate, phenylalanine, and glutamine together with the sulfone dimethylsulfone and the ketoacid pyruvate (Figure S5). Beta-hydroxybutyrate and acetoacetate decreased during the treatment period (Figure S5). All lipoprotein subfractions increased during the treatment. This impact was most strongly seen for VLDL subfractions (Figure S6)

### 3.4. Treatment Effects in Different Prognostic Relevant Cohorts

**Figure 5:**
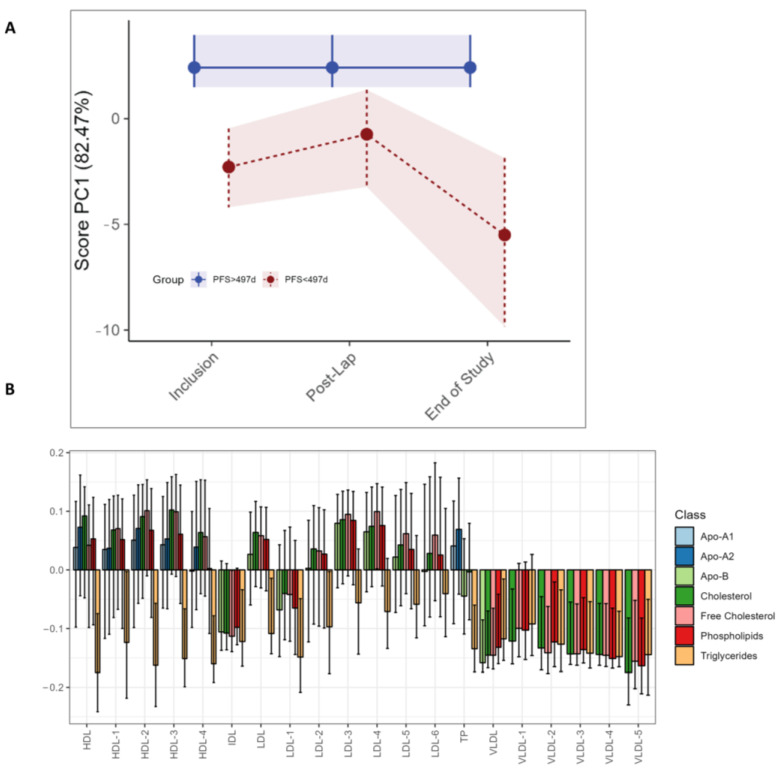
RM–ASCA+ scores plot of the time:group effect for PFS subgroups, demonstrating changes in lipoproteins over time. Only the three visits are included due to an uneven distribution of short vs. long PFS patients. The pre- and post-operative and pre-chemotherapy visits (Arm I only) had too few patients with short PFS to make a comparison. (**A**) The trajectory of lipoprotein subfractions (scores) on principal component 1 is shown for the short PFS group (red) relative to the long PFS (blue) group. The time development of the long PFS group has been removed to highlight the effect of short PFS. (**B**) The loading plot of lipoproteins shows the trajectory lines in (**A**), where the variation reflects differences in the short PFS group relative to the long PFS group. The scores show the overall development of lipoprotein subfractions over time. Bars are color coded according to the major subclasses of lipoproteins (Table S2). The 95% confidence interval is marked with black lines within the bars.

The LMM analysis demonstrates higher levels of phenylalanine (*p* < 0.001) after the diagnostic laparoscopy in the long PFS group, as was seen in the study as a whole, but no significant univariate LMM differences between the long and short PFS groups (Table S4). No differences in circulating metabolites were found with RM–ASCA+ (data not shown). For the lipoprotein subfractions, RM–ASCA+ demonstrates higher triglyceride levels in the short PFS group at all time points (Figure 5C). Additionally, higher levels of all VLDL subfractions are evident at all time points but particularly at the end of study in patients with shorter PFS (Figure 5A,C). The same differences in lipoprotein subfractions between long and short PFS patients are not observed by separate individual lipoprotein assessment by LMM (Table S4).

**Figure 6:**
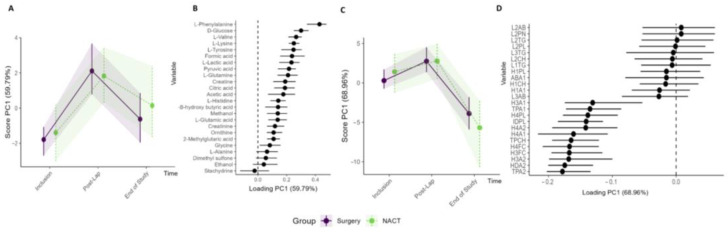
Subgroup analysis of Arm I (n = 15) primary cytoreductive surgery and Arm II (n = 9) NACT. RM–ASCA+ analysis showing the trajectories of metabolites (**A**,**B**) and lipoproteins (**C**,**D**) grouped into Arm I (purple) and Arm II (green). The inclusion, post-laparoscopy, and end-of-study time points are included in the analyses. Only the lipoprotein subfractions with the highest contribution to the PC1 are shown.

Analysis was performed to compare the circulating metabolite and lipoprotein profiles of the patients stratified to primary cytoreductive surgery (n = 15) versus NACT (n = 9). Both study groups showed similar changes in their metabolites and lipoprotein subfractions during the treatment period with no significant differences (Figure 6, Table S5, Figure S7). Furthermore, when comparing patients with RM–ASCA+ with and without residual tumor after primary cytoreductive surgery (R0 versus R ≠ 0) (Figure S8) or patients who received olaparib preoperatively versus standard-of-care treatment (Arm IA vs. Arm IB), no clinically relevant changes were identified.

## 4. Discussion

In the present study, we demonstrate that the serum metabolic and lipoprotein patterns change throughout the primary treatment for advanced HGSOC. While the circulating metabolites demonstrate reversible changes related to the surgical procedures, the lipoprotein subfractions more strongly associate with tumor-specific treatment. Significant associations toward a more atherogenic lipid profile with higher cholesterol levels were found after chemotherapy, indicating both an enhanced systemic inflammation and an increased risk of cardiovascular disease after treatment. An atherogenic lipid profile consists of elevated triglycerides and small-dense low-density lipoproteins together with low levels of high-density lipoprotein cholesterols [38]. Further, patients with an early disease relapse exhibited an even more atherogenic profile with significantly higher triglycerides and VLDL at the end of treatment together with lower HDL and LDL lipoprotein subfractions.

The complex tumor–host interactions can be explored through the longitudinal analysis of lipoproteins and metabolites, which reflect both the disease and the patient as well as the overall treatment response [20]. We demonstrate that toward the end of the treatment period, increasing levels in almost all lipoprotein parameters are evident, including total cholesterol (TPCH), Apolipoprotein-A1 and A2 (TPA1 and TPA2), Apo-A1 HDL, free cholesterol HDL, phospholipids IDL, and triglycerides IDL. These lipoproteins were also increased in the LMM analysis results (Table S3, Figures S5 and S6). Such changes have not been described for HGSOC previously, but are in line with a similar study of breast cancer patients [21]. The overall increase in total cholesterol level contributes to the downregulation of important immunological response mechanisms through T-cell exhaustion and activated myeloid-derived suppressor cells [39,40,41,42,43]. Our characterization of multiple lipoprotein fractions gives a broader assessment of the heterogeneous lipoprotein families. This is demonstrated with the elevated subfraction free cholesterol HDL, which takes part in the lipid transport to HDL and is regarded as a contributor of an atherogenic lipid profile even though HDL molecules in general are anti-atherogenic and anti-inflammatory. The increased levels of the Apo-A2 subfraction determine the HDL particle size and influence the serum concentration of cholesterol. Apo-A1 is a major component of HDL, but although increased Apo-A1 is regarded as an indicator for HDL cholesterol levels and hence protective against cardiovascular disease, the Apo B/Apo-A1 ratio seems to be important for cardiovascular risk prediction [44]. Triglyceride-rich lipoproteins appear to promote atherogenesis independently of LDL. This subset of lipoproteins promote a pro-atherogenic response by an enhanced recruitment of inflammatory proteins and by suppressing the anti-inflammatory effects of HDL. The similar treatment effects demonstrated in Arm I and Arm II (Figure 6) suggest that the atherogenic shift occurs irrespective of surgical treatment regimen and is associated with chemotherapy use. As the study period is 180 days (+/− 40) only, the probability of weight gain and inactivity influencing the results are low. In total, 16/19 of our patients experienced weight loss from inclusion to the end of treatment visit and only 2/19 a very modest weight gain. It is also unlikely that the intake of cholesterol-lowering drugs explains our findings as the six patients who reported statin use at baseline are equally distributed across the study arms and PFS groups. Whether the changes persist over time or only are transitional requires further investigation.

In the present study, we explored and compared prognostic biomarkers in patients with long and short PFS. Baseline associations revealed a pattern where a subset of patients with long PFS (n = 5) show higher levels of HDL and lower VLDL and a group of patients (n = 5) with short PFS show higher VLDL and lower HDL (Figure 3). Further, the RM–ASCA+ model demonstrated higher triglyceride lipoprotein subfractions at all time points in the short PFS group. Together with elevated cholesterols, elevated triglyceride levels can increase the risk for malignancies [45,46,47,48]. These increases combined with lower triglycerides in the long PFS group indicate that differences in inflammatory responses in patients could be associated with time to recurrence. Interestingly, for the end of treatment visit we demonstrated higher levels of the circulating metabolites dimethylsulfone and histidine, both activators of the innate immune system and thereby preventors of oncogenic immune escape [49,50,51]. This difference was related to the study visit and not to the PFS subgroups in the multivariate model.

The strong impact of surgery on metabolite and lipoprotein composition is unmistakable (Figure 4). The surgical treatment, either laparoscopy or primary cytoreductive surgery, represents a mechanical trauma that is known to cause metabolic, endocrine, and immunological changes [52]. The metabolites show a similar imprint after the two surgical procedures with increased levels of phenylalanine and tyrosine, two metabolites essential for catecholamine production, compounds important in stress responses [53]. For the lipoprotein subfractions, however, no changes are seen after the diagnostic laparoscopy. At the post surgery visit (Figure 4A,C), in which the tumor and organs are removed, and the duration of the operation is considerably longer, the LMM analysis demonstrated reduced levels of 42 lipoprotein subfractions, while four subgroups of VLDL5 increased. The latter is likely part of an activated innate immune response related to the surgical trauma. Metabolite and lipoprotein alterations could also emerge from the tumor load reduction although the same alterations are not seen after tumor-reducing NACT. This could indicate that the impact of surgery goes beyond tumor reduction alone or that the chemotherapeutic tumor reduction influences the metabolome differently than surgery.

The presence of residual malignant cells after surgical treatment constitutes an important clinical challenge in EOC treatment, and complete cytoreductive surgery remains the most important prognostic factor for survival. NACT is the preferred choice of treatment when complete cytoreductive surgery is unfeasible, but the surgical outcome is hard to predict. Our analysis failed to demonstrate any differences in the pretreatment and longitudinal metabolome between the groups with different surgical outcomes. This could be due to the limitations of our study: the included cohort is relatively small, the three study arms ended up being unequally distributed, and the serum sampling ended after three rounds of chemotherapy, when patients were radiologically and clinically evaluated for treatment. Another challenge is the selection of patients to primary cytoreductive surgery. Despite a thorough pre-operative evaluation, including a diagnostic laparoscopy, only 50% of the patients who were allocated to primary cytoreductive surgery were able to obtain the predicted complete cytoreductive surgery.

## 5. Conclusions

This is the first study describing the metabolic and lipoprotein changes that take place during primary treatment for HGSOC, and the results demonstrate a shift in the measurable circulating metabolites and lipoprotein subfractions according to the therapy given and can potentially be applied as a biomarker for risk of recurrence. The longitudinal sampling gives us exclusive insight into how our interventions affect the ultimate endpoint in the omics-cascade. In this study, we have demonstrated a shift toward a more atherogenic and inflammatory profile in lipoprotein subfractions irrespective of study arm and surgical outcome. Furthermore, the findings indicate that molecular phenotyping would be required in addition to clinical knowledge for better definition of prognostic subtypes. We suggest that future trials in patients with HGSOC should include metabolomic analyses to confirm these findings and further explore the increased risk for cardiovascular events during and after ovarian cancer treatment, which may demand further attention.

## Data Availability

The data that support the findings of this study are available on request from the corresponding author. The data are not publicly available due to ethical restrictions.

## Supplementary Materials

The following supporting information can be downloaded at https://www.mdpi.com/article/10.3390/metabo13030417/s1, Figure S1: Study design and allocation of patients to different subgroups; Figure S2: (A) overview of metabolites and lipoprotein subfractions analyzed; Figure S3: Metabolites—all values over time using ASCA-RM+. Figure S4: Metabolites and lipoproteins. Distribution in the two PFS groups; Figure S5: ASCA-RM+ analysis of metabolite changes during the treatment period; Figure S6: ASCA-RM+ analysis of lipoprotein changes during the treatment period; Figure S7: PLS-DA analysis performed for time of inclusion; Figure S8: Subgroup analysis of patients who obtained complete cytoreductive surgery (brown) and those who obtained suboptimal cytoreductive surgery (yellow) with trajectory lines (A) and loading plots; Figure S9: Metabolites from all patients, all visits included; Figure S10: Loadings from PCA plots; Table S1: patient characteristics; Table S2: Overview of lipoproteins in the NMR panel; Table S3: LMM analysis of serum metabolites and lipoproteins in all patients (n = 24) using samples collected at inclusion as a reference; Table S4: LMM analysis of patients where the subgroup long progression-free survival (reference group) values are tested in relation to short progression-free survival at each visit. Only time effects were significant in LMM; there were no group or time:group effects; Table S5: LMM analysis of patients where the subgroup Arm I (reference group) is tested in relation to Arm II at each visit.
