# Supplementary material for: Primary Treatment Effects for High-Grade Serous Ovarian Carcinoma Evaluated by Changes in Serum Metabolites and Lipoproteins
Source: Metabolites. 2023 Mar 12;13(3):417. doi: 10.3390/metabo13030417 (PMC10053757; doi:10.3390/metabo13030417)
Supplement: Supplementary file 1 [file metabolites-13-00417-s001.zip › Supplementary_material_Torkildsen_2023.pdf]

Primary treatment effects for high-grade serous ovarian carcinoma evaluated by changes in serum metabolites and lipoproteins

Cecilie Fredvik Torkildsen [1,2], Marie Austdal [3], Ann-Charlotte Iversen [4,5], Tone Frost Bathen [6], Guro Fanneløb Giskeødegård [7], Elisabeth Berge Nilsen [1], Grete Alræk Iversen [8], Ragnar Kvie Sande [1,9], Line Bjørge[2,8] and Liv Cecilie Vestrheim Thomsen [2,8,*]

[1] Department of Obstetrics and Gynecology, Stavanger University Hospital, 4068 Stavanger, Norway; cecilie.torkildsen@uib.no (C.F.T.)
[2] Centre for Cancer Biomarkers CCBIO, Dept of Clinical Science, University of Bergen, 5020 Bergen, Norway
[3] Department of Research, Stavanger University Hospital, 4068 Stavanger, Norway
[4] Centre of Molecular Inflammation Research (CEMIR) and Department of Clinical and Molecular Medicine, Norwegian University of Science and Technology (NTNU), 7491 Trondheim, Norway
[5] Department of Gynecology and Obstetrics, St. Olavs Hospital, Trondheim University Hospital, 7006 Trondheim, Norway
[6] Department of Circulation and Medical Imaging, Norwegian University of Science and Technology, 7491 Trondheim, Norway
[7] K.G. Jebsen Center for Genetic Epidemiology, Department of Public Health and Nursing, Norwegian University of Science and Technology (NTNU), 7491Trondheim, Norway
[8] Department of Obstetrics and Gynecology, Haukeland University Hospital, 5021 Bergen, Norway
[9] Department of clinical science, University of Bergen, 5020 Bergen, Norway
* Correspondence: liv.vestrheim@uib.no (L.C.V.T.)

Supplementary Methods

The IMPACT trial: Inclusion and exclusion criteria

Exclusion criteria were the inability to receive oral medication, allergies to the study drugs, recent treatment for another malignancy, active liver disease or HIV, and/or the inability to understand a written informed consent document.

Lipoprotein metabolite analysis using NMR

The density range of lipoprotein subfractions is continuous, and the subfractions referred to in this manuscript correspond to the density ranges as defined in protocols from Bruker BioSpin: LDL1: 1.019-1.031 kg/L, LDL2: 1.031-1.034 kg/L, LDL3: 1.034-1.037 kg/L, LDL4: 1.037-1.040 kg/L, LDL5: 1.040-1.044 kg/L, and LDL6: 1.044-1.063 kg/L. HDL1: 1.063-1.100 kg/L, HDL2: 1.100-1.112 kg/L, HDL3: 1.112-1.125 kg/L, and HDL4: 1.125-1.210 kg/L.

NMR buffer composition

The serum buffer was prepared as follows: i) Dissolved 10.05 g Na2HPO4•7H2O in 380 mL H2O; ii) added 0.4 g TSP; iii) mixed well by ultrasonic mixing; iv) added 5 mL of a 4% NaN3/H20 solution; v) adjusted pH to 7.4 with 1M HCl (1M NaOH); vi) added H2O until total volume is 400 mL; vii) added 100 mL D2O and mixed well.

Differences between the different sites of sampling

The patients were recruited from two sites: Haukeland University Hospital, Bergen, Norway and Stavanger University Hospital, Stavanger, Norway. Potential systematic differences between the two sites were explored by LMM analyses and RM-ASCA analyses of the metabolites and lipoproteins at all time points. No significant differences were found (data not shown).

RM-ASCA+ analysis, extended information

RM-ASCA+ allows the effect matrices from LMM to be analyzed either separately or combined. Alternatively, the effect matrix combining group+time*group interaction can be analyzed to display possible baseline differences between the groups together with their development over time compared to the reference group. An effect matrix combining "time+group+time*group interaction" will show the time development of all groups, including the reference group, and display possible baseline differences between them in one plot.

Supplementary Tables

Table S1. Patient characteristics.

	**All (n = 24)**	**Arm I (n = 15)**	**Arm II (n = 9)**	**p-value (Arm I vs. Arm II)**
Age in years	67.3 (54-85)	64.2 (54-78)	72.6 (56-85)	0.045
Stage (FIGO 2014)<br>Stage 2<br>Stage 3<br>Stage 4	 1<br>17<br>6	 1<br>11<br>3	 0<br>6<br>3	0.435
gBRCA mut (% tested)	1 (96%)	0 (100%)	0 (89%)	
sBRCA mut (% tested)	0 (46%)	0 (53%)	0 (33%)	
NACT	9	0	9	
Primary surgery<br>R0<br>R1<br>R2	15/24<br>7<br>1<br>7	 7<br>1<br>7	 NA<br>NA<br>NA	
BMI (m2/kg)	25 (18-32)	25 (21–26)	23 (18-27)	0.404
CA125 (kU/L)	876 (34-2332)	897 (34–2332)	837 (138-1372)	0.471
ECOG Score:<br>ECOG 0<br>ECOG 1<br>ECOG2	 11<br>11<br>2	 10<br>5<br>0	 1<br>6<br>2	0.002
Surgical complexity score	3.1 (0-6)	3.9 (1-7)	1.6 (0-3)	0.001
Comorbidities (%)<br>None<br>Cardiovascular<br>Other	 8<br>5<br>11	 7<br>3<br>5	 1<br>3<br>5	0.184
Cholesterol-reducing drug (%)<br>Yes<br>No	 6<br>18	 4<br>11	 2<br>7	0.603
Albumin (g/L)	38.8	40.4	36.2	0.170
Hemoglobin (g/dL)	13.2	13.7	12.2	0.017
Platelets (10**9/L)	387	342	461	0.079
Evaluation[a] (visit 13)<br>Complete response<br>Partial response<br>Stable disease<br>N/A<br>Death	 9<br>11<br>1<br>2<br>1	 8<br>6<br>1<br>0<br>0	 1<br>6<br>0<br>1<br>1	0.068
PFS (months) % reached	19<br>14.9 (1-39)	11<br>17.6 (7-37)	8<br>10.1 (1-16)	0.006
OS (months) % reached	8<br>22 (1-41)	3<br>31 (18-40)	5<br>14 (1-27)	0.089

Data are shown as number of patients in the respective cohorts otherwise as mean (min-max). PCS; Primary cytoreductive surgery, FIGO 2014; The international Federation of Gynecology and Obstetrics staging consensus from 2014, gBRCA mut; genetic BRCA 1/2 mutation, sBRCA mut; somatic BRCA 1/2 mutation, R0; Complete cytoreductive surgery (no residual tumor tissue after surgery), R1; Optimal cytoreductive surgery (residual tumor ≤ 1 cm), R2; Suboptimal cytoreductive surgery (residual tumor > 2 cm), ECOG; Eastern Cooperative Oncology Group Performance status, PFS; Progression-free survival, OS; overall survival, NA; not applicable.

*RECIST criteria supplemented by CA125 response and progression criteria developed by the Gynecologic Cancer InterGroup. CA125 progression has been integrated with objective criteria into a composite definition of progression often used in the frontline setting.

Table S2. Overview of lipoproteins in the NMR panel.

**Main parameters**	Unit	
TPTG	mg/dL	Triglycerides
TPCH	mg/dL	Cholesterol
LDCH	mg/dL	LDL Cholesterol
HDCH	mg/dL	HDL Cholesterol
TPA1	mg/dL	Apo-A1
TPA2	mg/dL	Apo-A2
TPAB	mg/dL	Apo-B100
**Calculated figures**		
LDHD		LDL Cholesterol / HDL
ABA1		Apo-A1/Apo-B100
TBPN		Total ApoB Particle number
VLPN	nmol/L	VLDL particle number
IDPN	nmol/L	IDL particle number
LDPN	nmol/L	LDL particle number
L1PN	nmol/L	LDL-1 particle number
L2PN	nmol/L	LDL-2 particle number
L3PN	nmol/L	LDL-3 particle number
L4PN	nmol/L	LDL-4 particle number
L5PN	nmol/L	LDL-5 particle number
L6PN	nmol/L	LDL-6 particle number
**Lipoprotein main fractions**		
VLTG	mg/dL	Triglycerides, VLDL
IDTG	mg/dL	Triglycerides, IDL
LDTG	mg/dL	Triglycerides, LDL
HDTG	mg/dL	Triglycerides, HDL
VLCH	mg/dL	Cholesterol, VLDL
IDCH	mg/dL	Cholesterol, IDL
VLFC	mg/dL	Free cholesterol, VLDL
IDFC	mg/dL	Free cholesterol, IDL
LDFC	mg/dL	Free cholesterol, LDL
HDFC	mg/dL	Free cholesterol, HDL
VLPL	mg/dL	Phospholipids, VLDL
IDPL	mg/dL	Phospholipids, IDL
LDPL	mg/dL	Phospholipids, LDL
HDPL	mg/dL	Phospholipids, HDL
HDA1	mg/dL	Apo-A1, HDL
HDA2	mg/dL	Apo-A2, HDL
VLAB	mg/dL	Apo-B, VLDL
IDAB	mg/dL	Apo-B, IDL
LDAB	mg/dL	Apo-B, LDL
**VLDL subfractions**		
V1TG	mg/dL	Triglycerides, VLDL-1
V2TG	mg/dL	Triglycerides, VLDL-2
V3TG	mg/dL	Triglycerides, VLDL-3
V4TG	mg/dL	Triglycerides, VLDL-4
V5TG	mg/dL	Triglycerides, VLDL-5
V1CH	mg/dL	Cholesterol, VLDL-1

V2CH	mg/dL	Cholesterol, VLDL-2
V3CH	mg/dL	Cholesterol, VLDL-3
V4CH	mg/dL	Cholesterol, VLDL-4
V5CH	mg/dL	Cholesterol, VLDL-5
V1FC	mg/dL	Free Cholesterol, VLDL-1
V2FC	mg/dL	Free Cholesterol, VLDL-2
V3FC	mg/dL	Free Cholesterol, VLDL-3
V4FC	mg/dL	Free Cholesterol, VLDL-4
V5FC	mg/dL	Free Cholesterol, VLDL-5
V1PL	mg/dL	Phospholipids, VLDL-1
V2PL	mg/dL	Phospholipids, VLDL-2
V3PL	mg/dL	Phospholipids, VLDL-3
V4PL	mg/dL	Phospholipids, VLDL-4
V5PL	mg/dL	Phospholipids, VLDL-5
**LDL subfractions**		
L1TG	mg/dL	Triglycerides, LDL-1
L2TG	mg/dL	Triglycerides, LDL-2
L3TG	mg/dL	Triglycerides, LDL-3
L4TG	mg/dL	Triglycerides, LDL-4
L5TG	mg/dL	Triglycerides, LDL-5
L6TG	mg/dL	Triglycerides, LDL-6
L1CH	mg/dL	Cholesterol, LDL-1
L2CH	mg/dL	Cholesterol, LDL-2
L3CH	mg/dL	Cholesterol, LDL-3
L4CH	mg/dL	Cholesterol, LDL-4
L5CH	mg/dL	Cholesterol, LDL-5
L6CH	mg/dL	Cholesterol, LDL-6
L1FC	mg/dL	Free Cholesterol, LDL-1
L2FC	mg/dL	Free Cholesterol, LDL-2
L3FC	mg/dL	Free Cholesterol, LDL-3
L4FC	mg/dL	Free Cholesterol, LDL-4
L5FC	mg/dL	Free Cholesterol, LDL-5
L6FC	mg/dL	Free Cholesterol, LDL-6
L1PL	mg/dL	Phospholipids, LDL-1
L2PL	mg/dL	Phospholipids, LDL-2
L3PL	mg/dL	Phospholipids, LDL-3
L4PL	mg/dL	Phospholipids, LDL-4
L5PL	mg/dL	Phospholipids, LDL-5
L6PL	mg/dL	Phospholipids, LDL-6
L1AB	mg/dL	Apo-B LDL-1
L2AB	mg/dL	Apo-B LDL-2
L3AB	mg/dL	Apo-B LDL-3
L4AB	mg/dL	Apo-B LDL-4
L5AB	mg/dL	Apo-B LDL-5
L6AB	mg/dL	Apo-B LDL-6
**HDL subfractions**		
H1TG	mg/dL	Triglycerides, HDL-1
H2TG	mg/dL	Triglycerides, HDL-2
H3TG	mg/dL	Triglycerides, HDL-3

H4TG	mg/dL	Triglycerides, HDL-4
H1CH	mg/dL	Cholesterol, HDL-1
H2CH	mg/dL	Cholesterol, HDL-2
H3CH	mg/dL	Cholesterol, HDL-3
H4CH	mg/dL	Cholesterol, HDL-4
H1FC	mg/dL	Free Cholesterol, HDL-1
H2FC	mg/dL	Free Cholesterol, HDL-2
H3FC	mg/dL	Free Cholesterol, HDL-3
H4FC	mg/dL	Free Cholesterol, HDL-4
H1PL	mg/dL	Phospholipids, HDL-1
H2PL	mg/dL	Phospholipids, HDL-2
H3PL	mg/dL	Phospholipids, HDL-3
H4PL	mg/dL	Phospholipids, HDL-4
H1A1	mg/dL	Apo-A1, HDL-1
H2A1	mg/dL	Apo-A1, HDL-2
H3A1	mg/dL	Apo-A1, HDL-3
H4A1	mg/dL	Apo-A1, HDL-4
H1A2	mg/dL	Apo-A2, HDL-1
H2A2	mg/dL	Apo-A2, HDL-2
H3A2	mg/dL	Apo-A2, HDL-3
H4A2	mg/dL	Apo-A2, HDL-4

Table S3. LMM analysis of serum metabolites and lipoproteins in all patients (n = 24) using samples collected at inclusion as a reference.

Metabolite	Effect (time point)	Estimate	p-value (adj)
Phenylalanine	Post-laparoscopy	2.46	< 0.001
Glucose	Post-laparoscopy	1.67	< 0.001
Valine	Post-laparoscopy	1.29	0.002
Lysine	Post-laparoscopy	1.40	0.003
Glutamine	Post-laparoscopy	1.03	0.008
Tyrosine	Post-laparoscopy	1.56	0.012
Lactate	Post-laparoscopy	1.12	0.016
Pyruvate	Post-laparoscopy	1.54	0.037
Formate	Post-laparoscopy	1.76	0.038
Phenylalanine	Post-surgery	2.91	< 0.001
Tyrosine	Post-surgery	2.64	< 0.001
Creatine	Post-surgery	1.83	< 0.001
Glucose	Post-surgery	1.86	< 0.001
Pyruvate	Post-surgery	1.89	0.035
TPCH	Post-surgery	-1.60	< 0.001
TPA1	Post-surgery	-1.56	< 0.001
HDA1	Post-surgery	-1.49	< 0.001
L6FC	Post-surgery	-1.80	< 0.001
TPA2	Post-surgery	-1.59	< 0.001
LDPL	Post-surgery	-1.39	< 0.001
LDCH	Post-surgery	-1.36	< 0.001
HDA2	Post-surgery	-1.59	< 0.001
L5FC	Post-surgery	-1.42	< 0.001
HDCH	Post-surgery	-1.19	< 0.001
LDAB	Post-surgery	-1.32	< 0.001
LDPN	Post-surgery	-1.32	< 0.001
H3FC	Post-surgery	-1.38	< 0.001
L5PL	Post-surgery	-1.34	< 0.001
L5AB	Post-surgery	-1.28	< 0.001
L5PN	Post-surgery	-1.28	< 0.001
LDFC	Post-surgery	-1.17	< 0.001
L6PL	Post-surgery	-1.53	< 0.001
TBPN	Post-surgery	-1.27	< 0.001
TPAB	Post-surgery	-1.27	< 0.001
L6CH	Post-surgery	-1.46	< 0.001
L5CH	Post-surgery	-1.23	< 0.001
H2A2	Post-surgery	-1.65	< 0.001
H1CH	Post-surgery	-1.01	< 0.001
H4A1	Post-surgery	-1.12	< 0.001
H1FC	Post-surgery	-0.91	< 0.001
L5TG	Post-surgery	-1.13	< 0.001
V5CH	Post-surgery	1.36	< 0.001
IDPL	Post-surgery	-1.25	< 0.001
L1FC	Post-surgery	-1.23	< 0.001
V5FC	Post-surgery	1.57	< 0.001
H3A2	Post-surgery	-1.57	< 0.001
L6AB	Post-surgery	-1.31	< 0.001
L6PN	Post-surgery	-1.31	< 0.001
HDPL	Post-surgery	-1.07	0.001
L4FC	Post-surgery	-1.06	0.002
H2CH	Post-surgery	-0.93	0.003

H2FC	Post-surgery	-0.88	0.0034
H1A1	Post-surgery	-0.83	0.0034
H3CH	Post-surgery	-1.08	0.0040
H1PL	Post-surgery	-0.81	0.0054
H2A1	Post-surgery	-0.90	0.0129
V5PL	Post-surgery	1.10	0.0179
H4FC	Post-surgery	-0.97	0.0179
V5TG	Post-surgery	1.08	0.0179
H1A2	Post-surgery	-0.77	0.0255
Glutamate	Pre-chemotherapy	1.27	0.040
Alanine	End of study	1.37	0.002
Histidine	End of study	1.05	0.004
Creatinine	End of study	0.55	0.027
Methylglutarate	End of study	0.97	0.027
TPCH	End of study	1.01	0.0001 (< 0.001)
H3FC	End of study	1.02	0.0005 (< 0.001)
TPA2	End of study	1.04	0.0009 (< 0.001)
HDA2	End of study	1.05	0.001
H4A1	End of study	0.88	0.002
H4PL	End of study	1.03	0.003
H4A2	End of study	0.83	0.003
H4FC	End of study	0.99	0.004
HDFC	End of study	0.70	0.007
IDPL	End of study	0.93	0.010
H3A2	End of study	1.13	0.022
V1CH	End of study	1.01	0.029
V1TG	End of study	0.94	0.030
IDTG	End of study	1.01	0.036
H3A1	End of study	0.97	0.036
H4CH	End of study	0.78	0.045

p-values adjusted according to Benjamini-Hochberg. Only significant findings are shown (p-values < 0.05).

Table S4. LMM analysis of patients where the subgroup long progression-free survival (reference group) values are tested in relation to short progression-free survival at each visit. Only time effects were significant in LMM; there were no group or time:group effects.

**Metabolite**	**Effect (time point, group or time:group interaction)**	**Estimate (ref group)**	**p-value (adj)**
Phenylalanine	Post-laparoscopy	1.41	0.000
Dimethylsulfone	End of study	1.22	0.001
Histidine	End of study	1.06	0.046
TPCH	End of study	1.062	0.017

p-values adjusted according to Benjamini-Hochberg. Only significant findings are shown (p-values < 0.05).

Table S5. LMM analysis of patients where the subgroup Arm I (reference group) is tested in relation to Arm II at each visit. Only time effects were significant in LMM; there were no group or time:group effects.

**Metabolite**	**Effect (time point, group or time:group interaction)**	**Estimate (ref group)**	**p-value (adj)**
Phenylalanine	Post-laparoscopy	1.39	0.006

p-values adjusted according to Benjamini-Hochberg. Only significant findings are shown (p-values < 0.05).

Supplementary Figures

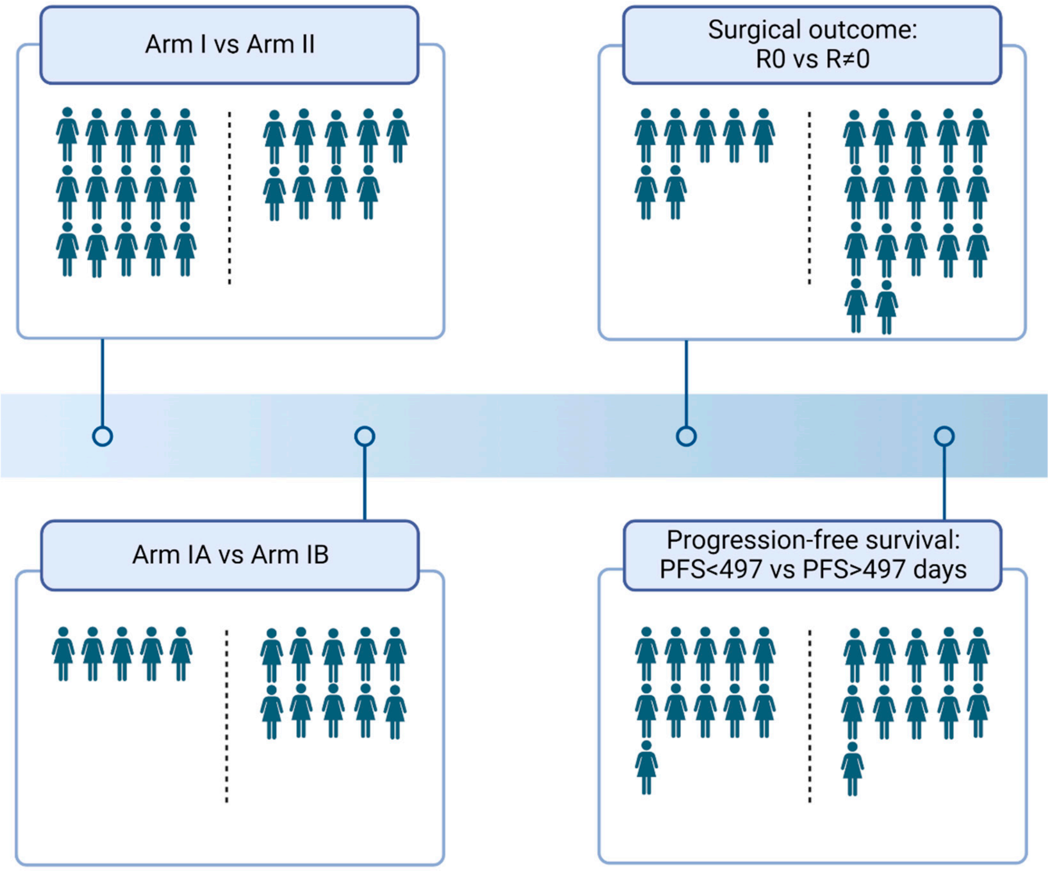

Figure S1. Study design and allocation of patients to different subgroups.

Arm I represents patients allocated to primary cytoreductive surgery after the diagnostic laparoscopy. Arm II consists of the patients allocated to neoadjuvant chemotherapy after the diagnostic laparoscopy. R0 represents patients in Arm 1 who obtained complete tumor resection at primary cytoreductive surgery. R≠0 represents patients in Arm I who did not obtain complete tumor resection at primary cytoreductive surgery and patients who underwent neoadjuvant chemotherapy (Arm II). Arm IA consists of patients who were allocated to PARP inhibitor treatment between the diagnostic laparoscopy and the cytoreductive surgery. Arm IB consists of patients who did not receive any additional treatment between the diagnostic laparoscopy and the primary cytoreductive surgery (standard-of-care treatment). Progression-free survival (PFS) was defined as the period from the date of trial inclusion to the diagnosis date of the first disease recurrence.

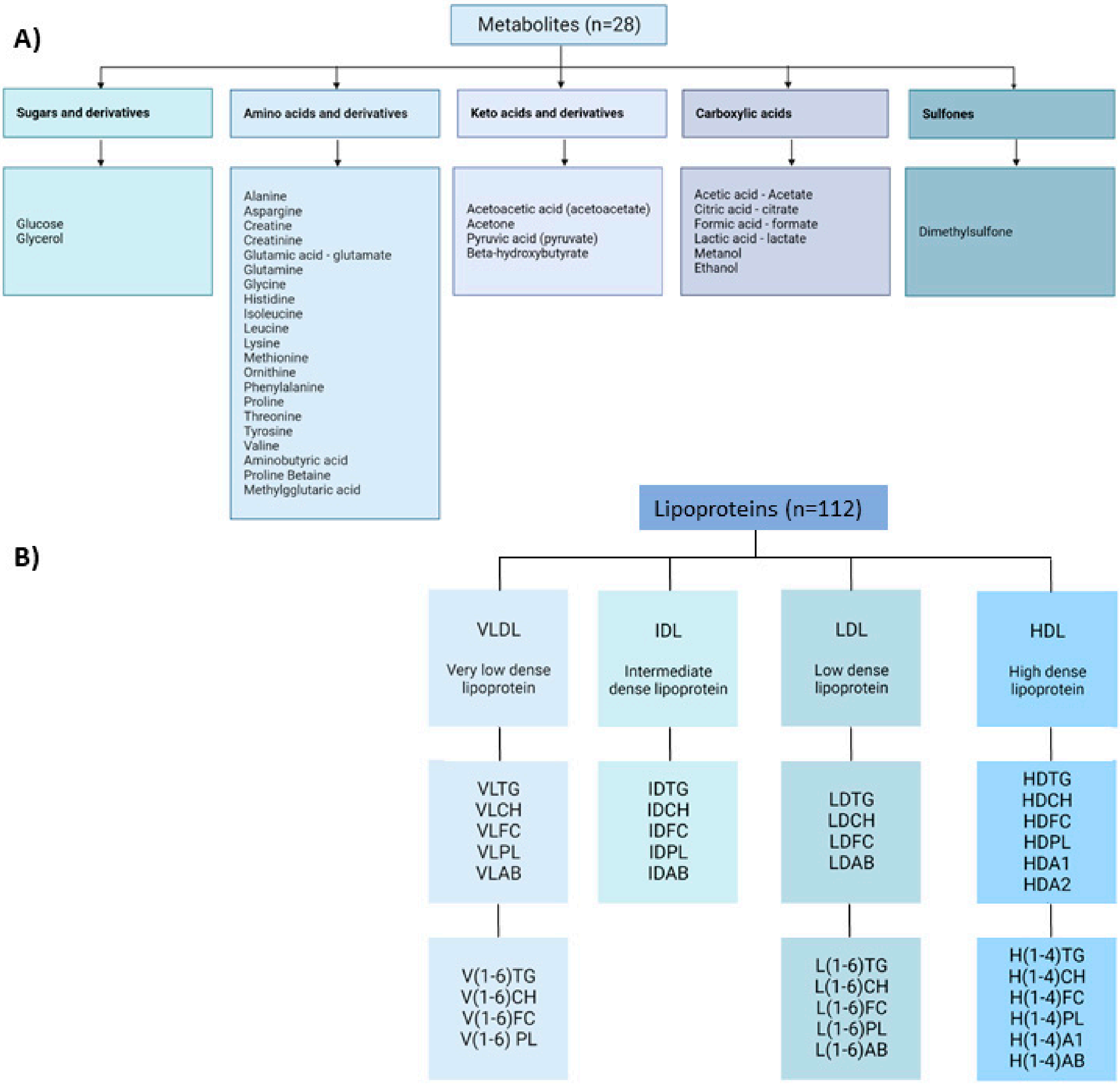

Figure S2. (**A**) Overview of metabolites (n = 28) and their subclasses. (**B**) Overview of lipoprotein subfractions. TG: triglycerides, CH: cholesterol, FC: free cholesterol, PL: phospholipids, AB: apolipoprotein B100, A1: apolipoprotein A1, A2: apolipoprotein A2.

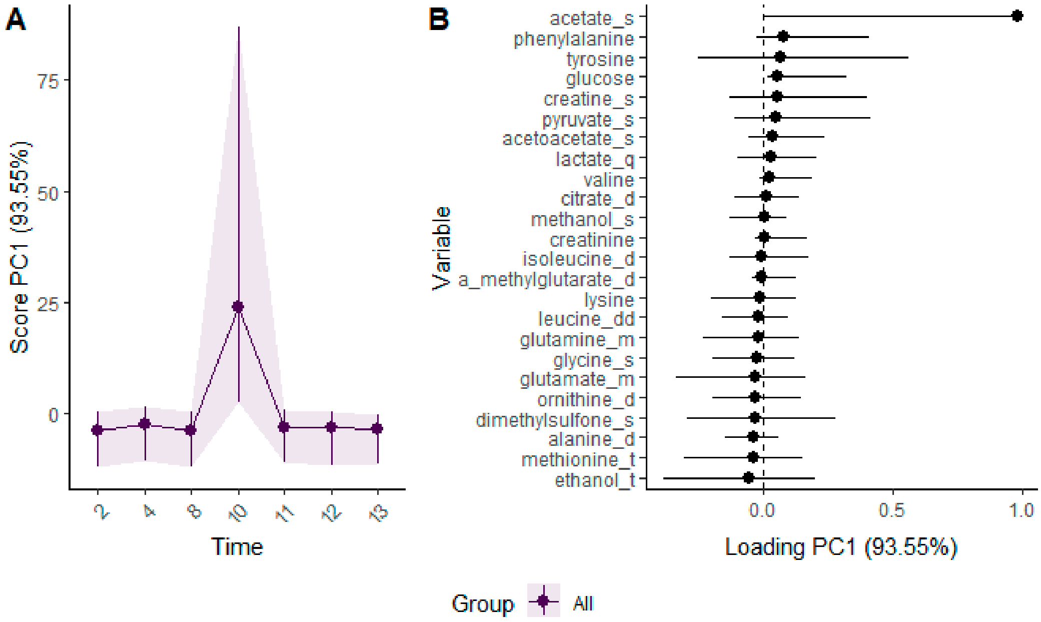

Figure S3. Metabolites—all values over time using ASCA-RM+. This analysis demonstrates a large increase in acetate at visit 10 (post-surgery) in one patient. Because it largely affects the model, we have imputed the single acetate value from this patient for the ASCA-RM+ plot in the figures in the main text.

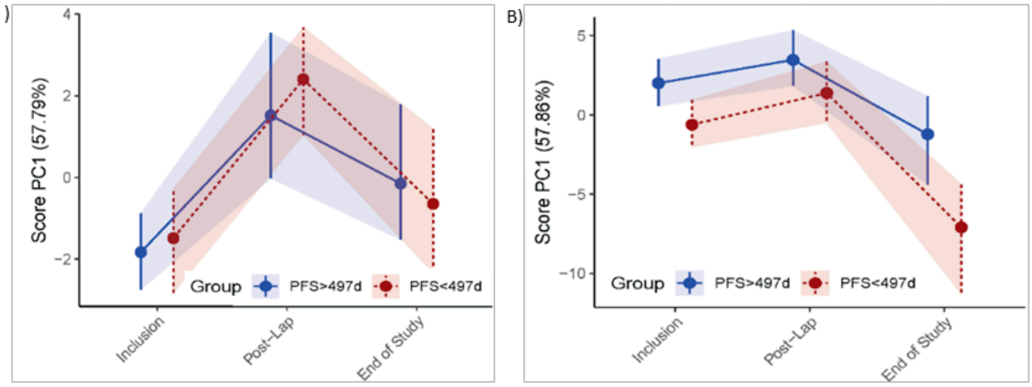

Figure S4. (**A**) Metabolites. Distribution in the two PFS groups. (**B**) Lipoproteins. Distribution in the two PFS groups.

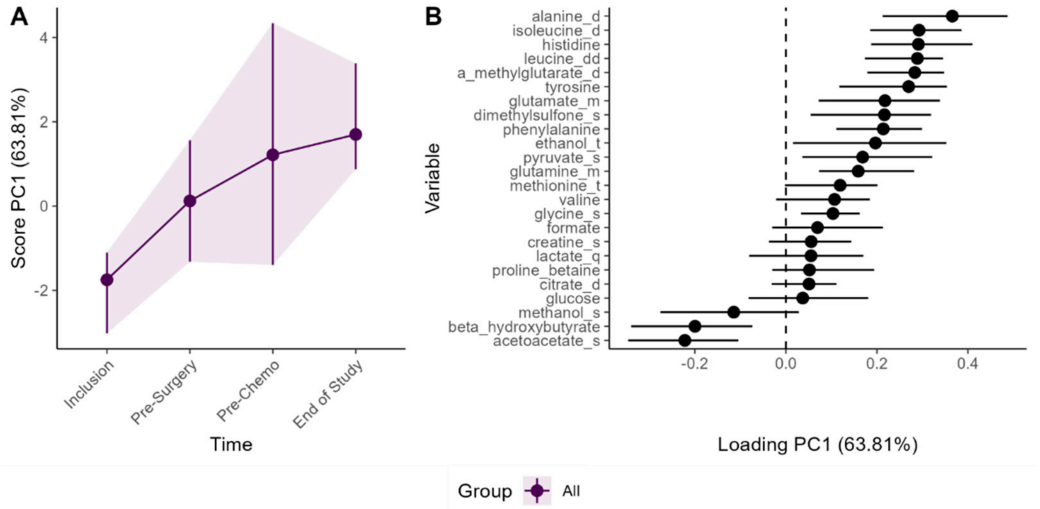

Figure S5. ASCA-RM+ analysis of metabolite changes during the treatment period. The ASCA-RM+ analysis of metabolite changes during the treatment period was performed without two visits: the post-laparoscopy and post-surgery visits.

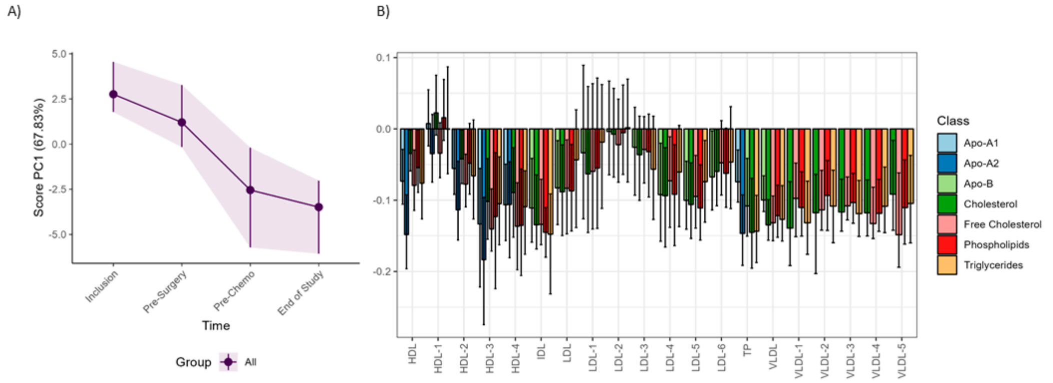

Figure S6. ASCA-RM+ analysis of lipoprotein changes during the treatment period. The ASCA-RM+ analysis of metabolite changes during the treatment period was performed excluding two visits: the post-laparoscopy and post-surgery visits.

Metabolite composition in patients who underwent surgery and chemotherapy (Arm I) vs. only chemotherapy (Arm II)

We performed an analysis of patients who had received neoadjuvant chemotherapy versus patients who had undergone primary surgery and received chemotherapy (last study visit) and found neither significant metabolic nor lipoprotein differences (**Figure S7**).

A subgroup analysis of the lipoprotein composition in patients with complete cytoreductive surgery vs. residual tumor or NACT treatment showed no significant differences (**Figure S7**). While phenylalanine, dimethylsulphone, creatinine, ethanol, glycine, and pyruvic acid increased in Arm II after the laparoscopy, the levels of β-hydroxybutyric acid and acetoacetic acid were decreased. The PLS-DA analysis did not identify significant differences in lipoproteins between the treatment arms after the operations. At the end-of-study visit, no differences could be demonstrated between these cohorts, neither for lipoproteins nor metabolite composition.

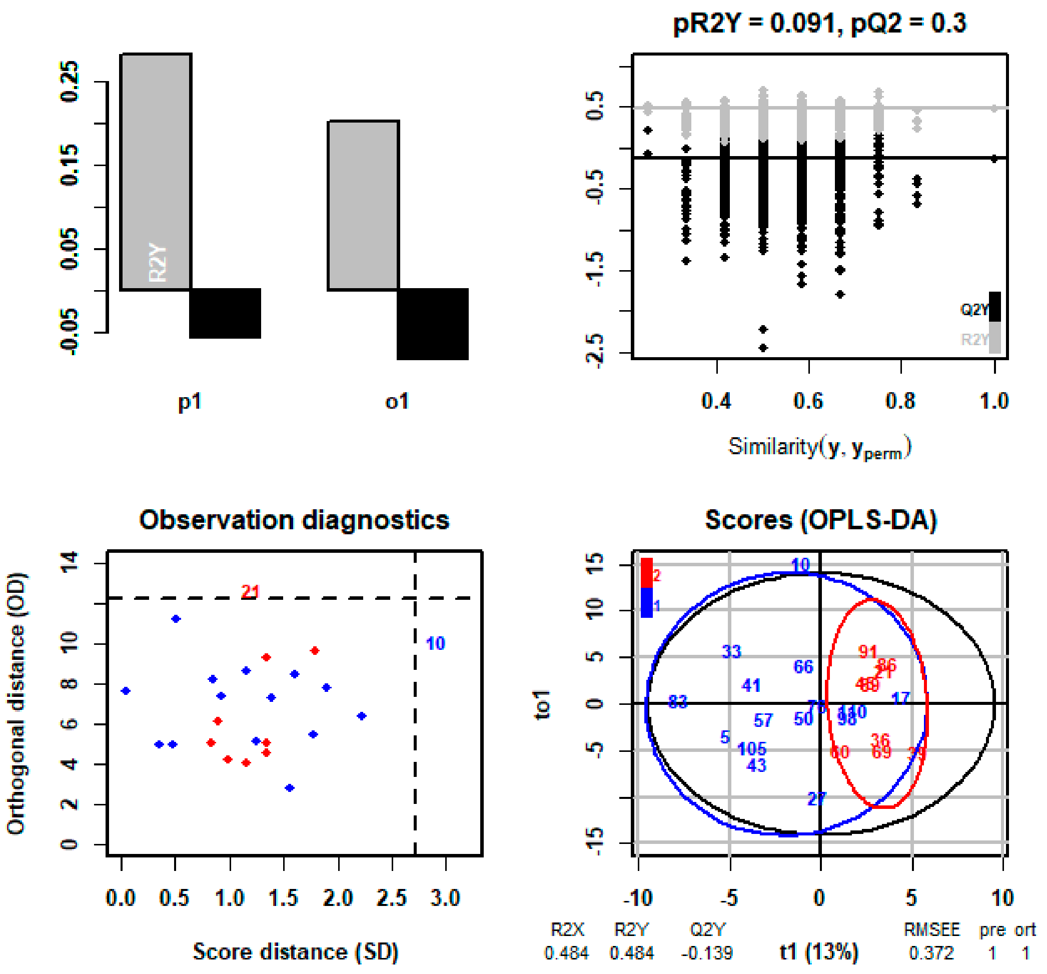

Figure S7. PLS-DA analysis performed for time of inclusion. The two trial arms were compared (Arm I vs. Arm II), and lipoproteins were applied as predictors.

Metabolite composition in patients who obtained complete cytoreductive surgery (R0) vs. patients with residual tumor or neoadjuvant chemotherapy (R≠0)

We explored metabolic and lipoprotein profiles between the two cohorts R0 (no residual tumor after surgery) and R≠0 (residual tumor after surgery) (**Figure S8**). This parameter was not associated with any robust differences in metabolite or lipoprotein composition. In the univariate analysis, we saw higher levels of glutamine, glutamate, and phenylalanine in patients who ended up with residual tumor, but these findings were not significant after multiple testing. In general, the LMM analysis demonstrated lower LDL-1, cholesterol, and free cholesterol at the end of treatment. Moreover, the ASCA-RM+ demonstrated the same tendency.

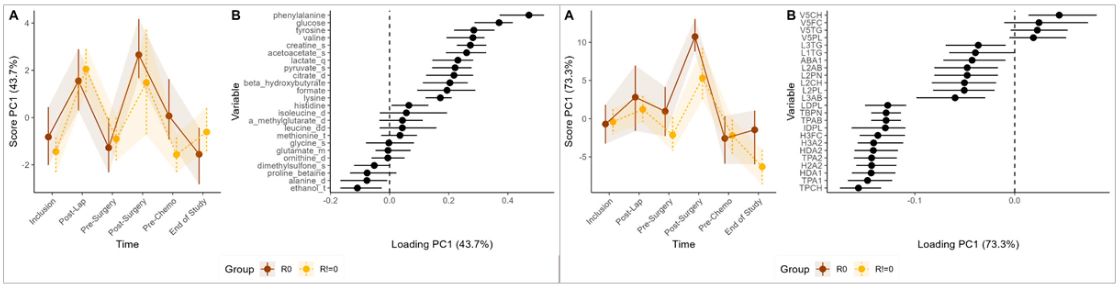

Figure S8. Subgroup analysis of patients who obtained complete cytoreductive surgery (brown) and those who obtained suboptimal cytoreductive surgery (yellow) with trajectory lines (A) and loading plots (B). To the left are metabolites and to the right lipoproteins. R0: successful surgery (n = 7); R≠0: residual tumor tissue after surgery (n = 16).

.

Arm IA vs. Arm IB (the impact of PARP inhibition)

Cohort IA received olaparib for 7–14 days prior to primary cytoreductive surgery. The number of patients tested for genomic or somatic BRCA mutations are outlined in Table S1. At inclusion, after olaparib treatment, and at the end of treatment, no differences in lipoprotein or metabolite composition were found (data not shown). Differences between the groups were found after the surgical procedures and after the cytoreductive surgery. Patients pre-treated with olaparib demonstrated increased phenylalanine, tyrosine, and glycine and decreased levels of 43 lipoproteins, including the main fractions cholesterol, Apo-A1, Apo-A2, LDL cholesterol, and HDL cholesterol. Samples taken before the initiation of chemotherapy showed decreased glutamate in the olaparib cohort and no differences in lipoproteins.

Metabolites

The ASCA-RM+ analyses fail to demonstrate an overall metabolic effect of treatment (A vs. B) (**Figure S9**). Arm IA has significantly lower phenylalanine ($p < 0.001$), glucose ($p = 0.027$), and lysine ($p = 0.029$) after the laparoscopic procedure. After the cytoreductive surgery, Arm IA exhibits lower levels of phenylalanine and tyrosine ($p < 0.001$ and 0.009, respectively) and higher levels of glycine ($p = 0.038$). Before the initiation of chemotherapy, glutamate is lower in the IA cohort ($p = 0.001$). At the end of study, the metabolite composition between these subgroups shows no differences.

Principal component analysis (PCA) plot of metabolites with lines between values for each patient

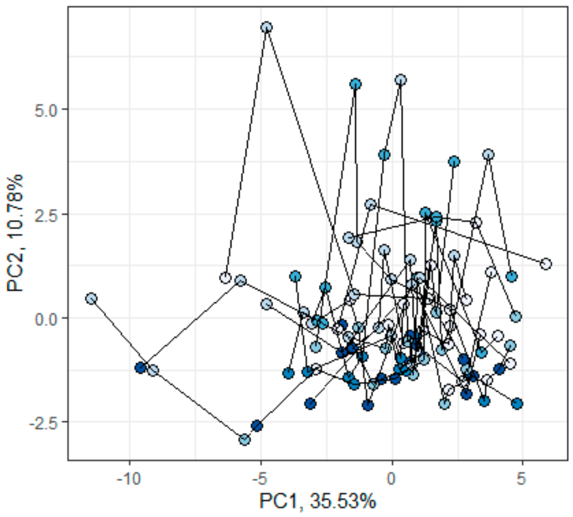

Figure S9. Metabolites from all patients, all visits included. The lines follow each individual patient during the study period.

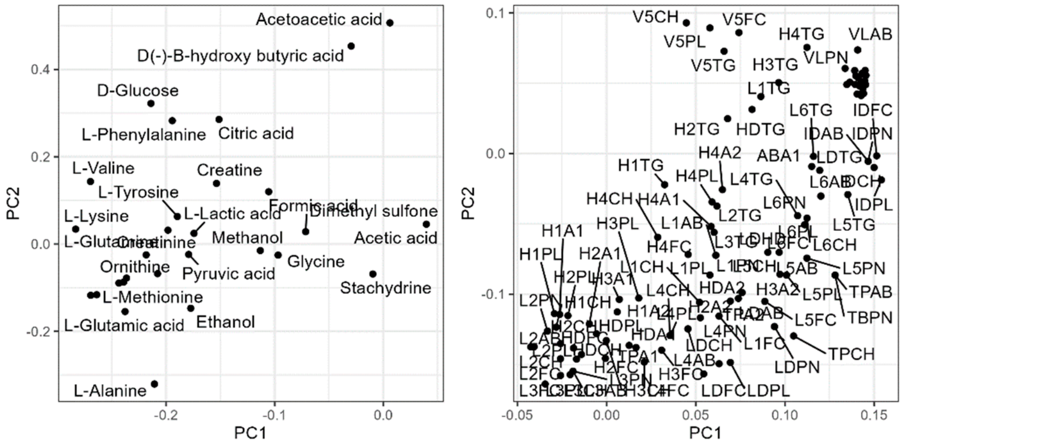

Figure S10. Loadings from the principal component analysis (PCA) plots in Figures 2A and 2B, showing the influence of the metabolites and lipoprotein variables on the PC scores.
